# One-Pot Self-Assembly of Dinuclear, Tetranuclear, and H-Bonding-Directed Polynuclear Cobalt(II), Cobalt(III), and Mixed-Valence Co(II)/Co(III) Complexes of Schiff Base Ligands with Incomplete Double Cubane Core

**DOI:** 10.3390/ma13235425

**Published:** 2020-11-28

**Authors:** Santokh S. Tandon, Neil Patel, Scott D. Bunge, Esther C. Wang, Rachel Thompson, Laurence K. Thompson

**Affiliations:** 1 Department of Chemistry and Biochemistry, Kent State University - Salem Campus, Salem, OH 44460, USA; 2 Department of Chemistry and Biochemistry, Kent State University, Kent, OH 44242, USA; neilnpatel89@gmail.com (N.P.); sbunge@kent.edu (S.D.B.); ewang2@kent.edu (E.C.W.); rthoma17@kent.edu (R.T.); 3 Department of Chemistry, Memorial University, St. John’s, NL A1B 3X7, Canada; laurie.thomp@gmail.com

**Keywords:** self-assembly of Co(II), Co(III), mixed-valence Co(II)/Co(III) complexes of Schiff base ligands, dinuclear, tetranuclear, polynuclear Co(II), Co(III), Co(II)/Co(III) complexes, mixed-valence Co(II)/Co(III) complexes, Cobalt(II)/Cobalt(III) complexes of Schiff base ligands with incomplete double cubane core, H-bonding-directed formation of cobalt supramolecular architectures with1D-single chains and 2D-sheet structures

## Abstract

The reaction of 2,6-diformyl-4-methylphenol (DFMF) with 1-amino-2-propanol (AP) and tris(hydroxymethyl)aminomethane (THMAM) was investigated in the presence of Cobalt(II) salts, (X = ClO_4_^−^, CH_3_CO_2_^−^, Cl^−^, NO_3_^−^), sodium azide (NaN_3_), and triethylamine (TEA). In one pot, the variation in Cobalt(II) salt results in the self-assembly of dinuclear, tetranuclear, and H-bonding-directed polynuclear coordination complexes of Cobalt(III), Cobalt(II), and mixed-valence Co^II^Co^III^: [Co_2_^III^(H_2_L^−^^1^)_2_(AP^−^^1^)(N_3_)](ClO_4_)_2_ (**1**), [Co_4_(H_2_L^−^^1^)_2_(µ_3_-1,1,1-N_3_)_2_(µ-1,1-N_3_)_2_Cl_2_(CH_3_OH)_2_]·4CH_3_OH (**2**), [Co_2_^II^Co_2_^III^(HL^−^^2^)_2_(µ-CH_3_CO_2_)_2_(µ_3_-OH)_2_](NO_3_)_2_·2CH_3_CH_2_OH (**3**), and [Co_2_^II^Co_2_^III^ (H_2_L1^2^^−^)_2_(THMAM^−1^)_2_](NO_3_)_4_ (**4**). In **1**, two cobalt(III) ions are connected via three single atom bridges; two from deprotonated ethanolic oxygen atoms in the side arms of the ligands and one from the1-amino-2-propanol moiety forming a dinuclear unit with a very short (2.5430(11) Å) Co-Co intermetallic separation with a coordination number of 7, a rare feature for cobalt(III). In **2**, two cobalt(II) ions in a dinuclear unit are bridged through phenoxide O and μ_3_-1,1,1-N_3_ azido bridges, and the two dinuclear units are interconnected by two μ-1,1-N_3_ and two μ_3_-1,1,1-N_3_ azido bridges generating tetranuclear cationic [Co_4_(H_2_L^−^^1^)_2_(µ_3_-1,1,1-N_3_)_2_(µ-1,1-N_3_)_2_Cl_2_(CH_3_OH)_2_]^2+^ units with an incomplete double cubane core, which grow into polynuclear 1D-single chains along the *a*-axis through H-bonding. In **3**, HL^2−^ holds mixed-valent Co(II)/Co(III) ions in a dinuclear unit bridged via phenoxide O, μ-1,3-CH_3_CO_2_^−^, and μ_3_-OH^−^ bridges, and the dinuclear units are interconnected through two deprotonated ethanolic O in the side arms of the ligands and two μ_3_-OH^−^ bridges generating cationic tetranuclear [Co_2_^II^Co_2_^III^(HL^−^^2^)_2_(µ-CH_3_CO_2_)_2_(µ_3-_OH)_2_]^2+^ units with an incomplete double cubane core. In **4**, H_2_L1^−2^ holds mixed-valent Co(II)/Co(III) ions in dinuclear units which dimerize through two ethanolic O (μ-RO^−^) in the side arms of the ligands and two ethanolic O (μ_3_-RO^−^) of THMAM bridges producing centrosymmetric cationic tetranuclear [Co_2_^II^Co_2_^III^ (H_2_L1^−^^2^)_2_(THMAM^−1^)_2_]^4+^ units which grow into 2D-sheets along the *bc*-axis through a network of H-bonding. Bulk magnetization measurements on **2** demonstrate that the magnetic interactions are completely dominated by an overall ferromagnetic coupling occurring between Co(II) ions.

## 1. Introduction

Using simple building blocks, living organisms use self-assembly processes to fabricate symmetrical biomolecules (proteins, DNA, lipids, enzymes), with varied levels of structural complexities [1,2,3]. Inspired by nature, during the last few decades, chemists have used self-assembly processes successfully [4,5,6] to generate a variety of supramolecular architectures (organic materials [7,8,9], metalacyclic polygons and polyhedral [10], and nanoscale systems [11,12]) with desired size, shape, and function. The self-assembly process often utilizes a variety of cooperative and noncovalent interactions such as hydrogen bonding, strong electrostatic, and van der Walls forces, π–π stacking, hydrophobic, hydrophilic, and metal–ligand interactions to produce complex supramolecular architectures which are difficult or in some cases impossible to make otherwise. Self-assembly processes have many advantages over the stepwise synthesis of large supramolecular assemblies, and lead to the formation of desired products from the predetermined building blocks spontaneously and more efficiently while minimizing/eliminating the formation of side products. In most of the self-assembly processes, metal ions or hydrogen ions (H^+^) are used as a template for producing supramolecular coordination complexes with 1D-, 2D-, and 3D-network structures and grid systems [13,14,15,16,17,18,19,20,21,22,23].

Over the past thirty-five years, we have been interested in the self-assembly of polynuclear coordination complexes of transition metals to get a deeper insight into magneto-structural relationships, to understand the role of metal ions in self-assembly and structural complexities of assemblies produced, and the effects of the anions on the formation and coordination abilities of the macrocyclic and non-cyclic Schiff base ligands. Our interest in this area originated from the implications of transition metal complexes in homogeneous catalysis [24,25], as enzyme models [26,27,28,29], and their potential applications in magnetic materials [30,31,32,33,34]. Specifically, polynuclear cobalt complexes have attracted much attention in recent years due to their role as catalysts for light-driven water oxidation and in the photocatalytic trifluoromethylation of polycyclic aromatic hydrocarbons [35,36]. We have used the self-assembly process very successfully for generating spin-coupled coordination complexes of transition metal ions with macrocyclic [23,37,38,39,40,41,42,43,44,45,46,47,48,49,50,51] and non-cyclic [52,53,54,55,56] Schiff base ligands. In addition to polynucleating multidentate ligands, a majority of the coordination complexes also involve doubly or triply bridging anions like N_3_^−^, OH^−^, O^2−^, CH_3_O^−^, NCS^−^, N(CN)_2_^−^, CN^−^, and C_2_H_3_O_2_^−^ C_6_H_5_O^−^ to generate extended network structures [57,58,59,60,61,62].

In continuation of our interest in the self-assembly, structural characterization, and magnetic properties of polynuclear transition metal and lanthanide coordination complexes exhibiting ferromagnetic and antiferromagnetic spin exchange interactions, we have explored the coordination versatility of the Schiff base ligands derived from the condensation of 2,6-diformyl-4-methylphenol (DFMP) with 2-aminoethanol, 1-amino-2-propanol, 2-amino-1,3-propanediol, and tris(hydroxymethyl)aminomethane with Copper(II) [52,54,55], Cobalt(II)/Cobalt(III) [53], and Nickel(II) [53,55,56]. These ligands have a high degree of conformational flexibility and the potential to coordinate in a convergent (directing the formation of the dinuclear units), and a divergent fashion (ability to extend coordination beyond the primary coordination mode), creating one-dimensional single chains, two-dimensional sheets, or three-dimensional network structures utilizing one, two, or three protonated/deprotonated hydroxymethyl/hydroxyethyl groups present in each side arm.

In this publication, we want to report the synthesis, structural characterization, spectroscopic studies, and magnetic properties of dinuclear Cobalt(III), tetranuclear Cobalt(II), and mixed-valence Cobalt(II)/Cobalt(III) complexes with an incomplete double cubane core, which grow into beautiful 1D-single chains and 2D-sheet structures through H-bonding-directed polymerization involving coordinated/uncoordinated hydroxy groups in the side arms of the ligands and methanol/water molecules/NO_3_^−^ ions in the crystal lattice. 

## 2. Materials and Methods

### 2.1. Physical Measurements

Exactly same as reported in recently published paper [56].

### 2.2. Materials

2,6-Diformyl-4-methylphenol (DFMP) was isolated by the reported method [63], and 1-amino-2-propanol (AP) and tris(hydroxymethyl)aminomethane (THMAM) were supplied by Aldrich. All other chemicals (solvents and metal salts) used were analytical or reagent grade and were used without further purification. Schiff base ligands used in this investigation have not been isolated and structurally characterized. They have been generated in situ by reacting DFMP with AP (H_3_L) and THMAM (H_4_L1) in the presence of the metal salts.

### 2.3. X-ray Crystallography

Suitable single crystals for X-ray diffraction studies were obtained for **1**–**4**. Crystal data for the compounds were collected by the same method by mounting a crystal onto a thin glass fiber from a pool of Fluorolube^TM^ and immediately placing it under a liquid N_2_ cooled stream, on a Bruker AXS diffractometer (Bruker AXS, Inc.: Madison, WI, USA) upgraded with an APEX II CCD detector (Bruker AXS, Inc.: Madison, WI, USA). The radiation used is graphite monochromatized Mo Kα radiation (λ = 0.7107 Å). The lattice parameters are optimized from a least-squares calculation on carefully centered reflections. Lattice determination, data collection, structure refinement, scaling, and data reduction were carried out using the APEX3 Version 2018.11 software (Bruker AXS, Inc.: Madison, WI, USA) package [64,65]. The data were corrected for absorption using the SCALE program within the APEX3 software (Version 6.45A, Bruker AXS, Inc.: Madison, WI, USA) package [64,65]. The structures were solved using SHELXT [66]. This procedure facilitated the assignment of C, N, Co, O, and Cl atoms. Subsequent Fourier synthesis yielded the remaining atom positions. The hydrogen atoms are fixed in positions of ideal geometry (riding model) and refined within the XSHELL software (Version 6.12, Bruker AXS, Inc.: Madison, WI, USA) package [67]. The final refinement of each compound included anisotropic thermal parameters on all non-hydrogen atoms and was performed using OLEX2-1.2 [68]. The crystal data for compounds **1**–**4** are given in Table 1. Crystallographic data for the structures have been deposited with the Cambridge Crystallographic Data Centre as supplementary publication nos: CCDC-1948320-1948323. Copies of the data can be obtained free of charge on application to CCDC, 12 Union Road, Cambridge CB2 1EZ, UK (fax, +44-(0)1223-336033; or e-mail, deposit@ccdc.cam.ac.uk). Selected interatomic distances and angles for compounds **1**–**4** are listed in the Tables S1–S4.

## 3. Results

### 3.1. Synthesis of Complexes

Caution: Azide and perchlorate complexes of metal ions involving organic ligands are potentially explosive. Only small quantities of the complexes should be prepared and should be handled with care.

In some cases, there is a difference between the most reasonable formula based on the elemental analysis (analytical formula) and that obtained from X-ray crystallography. This is because the analysis was carried out on air-dried samples due to their explosive nature. For consistency, the x-ray formulae will be used throughout this report.

### 3.2. [Co_2_^III^(H_2_L^−1^)_2_(AP^−1^)(N_3_)](ClO_4_)_2_ (***1***)

1-amino-2-propanol (AP) (2.0 mmol, 0.15 g) was dissolved in methanol (20 mL), and a solution of DFMP (1.0 mmol, 0.16 g) dissolved in hot methanol (20 mL) was added to it. The resultant yellow solution was stirred under reflux for 30 min. A solution of Co(ClO_4_)_2_∙6H_2_O (2.2 mmol, 0.82 g) dissolved in methanol (20 mL) was added to it while stirring under reflux dropwise over a period of 5 min. The reddish brown solution formed was refluxed further for 30 min, and a solution of sodium azide (2.0 mmol, 0.13 g) dissolved in a mixture of methanol:water (5:1 mL) was added dropwise. The dark red clear solution formed was stirred under reflux for 1 h and filtered while hot. A total of 5 mL of ethanol was added to the filtrate and was kept at room temperature for slow evaporation. After four weeks, reddish brown crystals suitable for X-ray analysis were obtained. The crystals were separated and washed with ethanol (2 × 2 mL). Yield, 0.14 g, 27.8%. IR spectrum: 3445, 3294, 3243, 3142 cm^−1^ (υ(OH or NH_2_) H_2_O/AP^−1^), 2027 cm^−1^ (υ_as_ (N_3_)), 1651 cm^−1^ (υ(C=N)), 1095, 1043, 995 cm^−1^ υ(ClO_4_^−^). UV–Vis Spectrum: 430, 315, and 270 nm (Co-azide and Co-ligand charge transfer transitions, respectively), and 525 nm (d–d transition). Elemental analysis (air-dried sample). Found (%): C, 36.48; H, 5.74; N, 10.63. Calcd. (%) for [Co_2_(C_15_H_21_N_2_O_3_)_2_(C_3_H_9_NO)(N_3_)] (ClO_4_)_2_·5H_2_O: C, 36.75; H, 5.70; N, 10.39.

### 3.3. [Co_4_(H_2_L^−1^)_2_(µ_3_-1,1,1-N_3_)_2_(µ-1,1-N_3_)_2_Cl_2_(CH_3_OH)_2_]·4CH_3_OH (***2***) 

Complex **2** was prepared by the same procedure as used for **1** except for replacing Co(ClO_4_)_2_·6H_2_O with CoCl_2_·6H_2_O (2.6 mmol, 0.62 g). After refluxing the reaction mixture for 2.5 h, a blood red clear solution formed was filtered while hot and the filtrate was kept at ambient temperature for slow evaporation. After 24 h, reddish brown crystals suitable for X-ray studies were formed. The crystals were separated and washed with ethanol (2 × 2 mL). Yield, 0.31 g, 53.7%. IR spectrum: 3374 cm^−1^ (υ(OH) H_2_O and/or CH_3_OH), 2084 cm^−1^ (υ_as_(N_3_)), 1648 cm^−1^ (υ(C=N)). UV–Vis Spectrum: 410, 390, and 310 nm (Co-azide and Co-ligand charge transfer transitions, respectively), and 680, 655, 605, 595 nm (d–d transition). Elemental analysis (air-dried sample). Found (%): C, 31.16; H, 5.28; N, 18.77. Calcd. (%) for [Co_4_(C_15_H_21_N_2_O_3_)_2_(CH_3_OH)_2_(N_3_)_4_Cl_2_]·7H_2_O: C, 31.50; H, 5.29; N, 18.38. 

### 3.4. [Co_2_^II^Co_2_^III^(HL^−2^)_2_(µ-CH_3_CO_2_)_2_(µ_3-_OH)_2_](NO_3_)_2_·2CH_3_CH_2_OH (***3***)

Complex **3** was obtained from a reaction carried out with the intention of getting a mixed metal (Gd/Co) complex but instead only a mixed-valence cobalt complex **3** was formed. To a methanolic solution (5 mL) of 1-amino-2-propanol (0.50 mmol, 0.040 g), a solution of DFMP (0.25 mmol, 0.040 g) dissolved in hot methanol (10 mL) was added. The resultant yellow solution of the Schiff base was stirred under reflux for 30 min. To it, a solution of Co(NO_3_)_2_∙6H_2_O (0.70 mmol, 0.200 g) in methanol (5 mL) was added dropwise, while stirring under reflux. This was followed by the addition of a solution of Gd(CH_3_CO_2_)_3_·XH_2_O (0.60 mmol, 0.200 g) in 5 mL of methanol. To the orange-red solution formed, a solution of triethylamine (TEA) (0.80 mmol, 0.080 g) was added dropwise, and the reaction mixture was stirred under reflux for 40 min. It was filtered while hot and the filtrate was left at room temperature for slow evaporation. After two weeks, the reddish brown residue left after the evaporation of the solvent was treated with 10 mL of ethanol. Most of the solid dissolved leaving behind some white solid which was discarded. After leaving the filtrate at room temperature for 10 weeks, reddish brown crystals suitable for X-ray analysis were formed. Some of the crystals were kept in the mother liquor for X-ray analysis and the rest were filtered off and washed with ethanol (2 × 1 mL). In this reaction, the presence of both CH_3_CO_2_^−^ and NO_3_^−^ ions or Gd^3+^ ion seems to play an important role for the formation of complex **3.** When this reaction was carried out with only Co(CH_3_CO_2_)_2_ or Co(NO_3_)_2_, the desired compound was not obtained. Yield, 0.115 g, 78.8%. IR spectrum: 3420 cm^−1^ (υ(OH) H_2_O and/or CH_3_CH_2_OH), 1647, 1637 cm^−1^ υ(C=N)/CH_3_CO_2_^−^). UV–Vis Spectrum: 400 nm and 310 nm (Co-ligand charge transfer transitions), and 550 nm (d–d transition). Elemental analysis (air-dried sample). Found (%): C, 35.79; H, 5.04; N, 7.34. Calcd. (%) for [Co_2_^II^Co_2_^III^(C_15_H_20_N_2_O_3_)_2_(CH_3_CO_2_)_2_(OH)_2_](NO_3_)_2_·CH_3_CH_2_OH·5H_2_O: C, 36.01; H, 5.37; N, 7.00.

### 3.5. [Co_2_^II^Co_2_^III^(H_2_L1^−2^)_2_(THMAM^−1^)_2_](NO_3_)_4_ (***4***)

Tris(hydroxymethyl)aminomethane (THMAM) (1.0 mmol, 0.12 g) was dissolved in 10 mL of methanol and a solution of DFMP (0.50 mmol, 0.080 g) in10 mL of hot methanol (10 mL) was added to it. The reaction mixture was stirred under reflux for 30 min. A solution of Co(NO_3_)_2_·6H_2_O, (1.2 mmol, 0.365 g) in 10 mL of methanol was added to it dropwise while stirring under reflux. The reddish brown solution formed was stirred under reflux for 15 min, and 10 drops of triethylamine (TEA) were added to it. A dark red solution formed was stirred under reflux for about 2.0 h and filtered while hot. The filtrate was left at ambient temperature for slow evaporation. After two weeks, reddish brown crystals suitable for X-ray analysis were formed. The crystals were filtered and washed with methanol (2 × 1 mL). Yield: 0.095 g, 34.4%. IR spectrum: 3333 cm^−1^, 3179 cm^−1^ (υ(OH/NH_2_) H_2_L1^2−^/THMAM), 1647cm^−1^, 1638 cm^−1^ υ(C=N/C=O). UV–Vis Spectrum: 440, 415, and 320 nm (Co-ligand charge transfer transitions), and 650, 525 nm (d–d transition). Elemental analysis (air-dried sample). Found (%): C, 35.78; H, 4.49; N, 9.73. Calcd. (%) for [Co_2_^II^Co_2_^III^(C_13_H_15_NO_5_)_2_(C_4_H_10_NO_3_)_2_](NO_3_)_4_: C, 35.93; H, 4.43; N, 9.86.

## 4. Discussion

### 4.1. Synthesis of the Complexes

**Figure 1:**
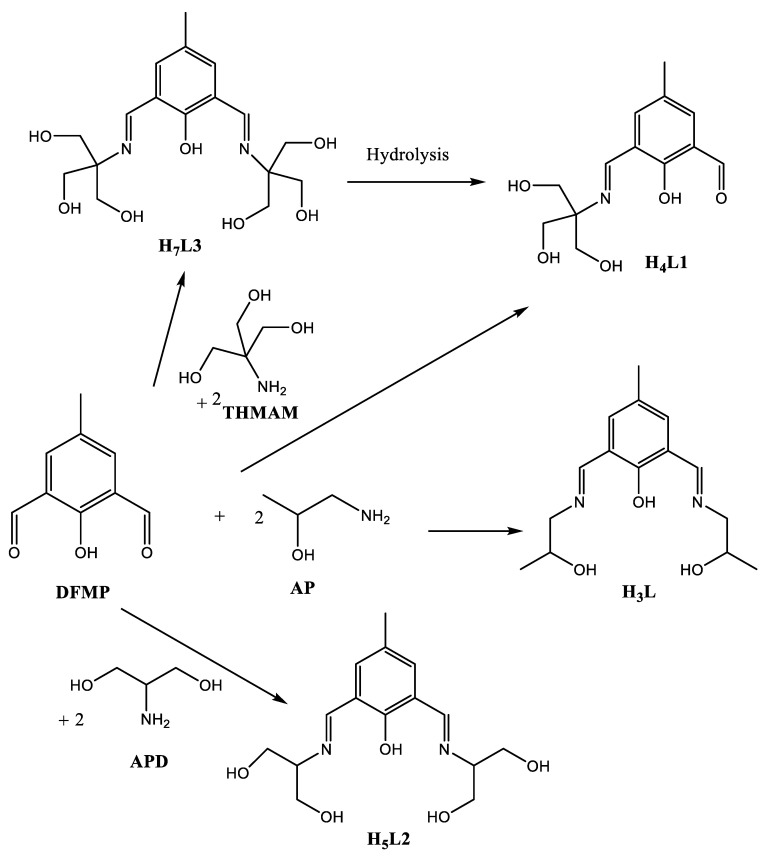
Structures of Schiff base ligands. DFMP = 2,6-diformy1-4-methylphenol, THMAM = tris(hydroxymethyl)aminomethane, AP = 1-amino-2-propanol.

In this publication, we wish to report the self-assembly, structural characterization, and magnetic properties of cobalt(II) (**2**), cobalt(III) (**1**), and mixed-valence (Co_2_^II^/Co_2_^III^) (**3** and **4**) complexes of two very versatile Schiff base: H_3_L (a double Schiff base, potentially pentadentate (N_2_O_3_) trianionic ligand) and H_4_L1 (a single Schiff base, potentially hexadentate (NO_5_), tetraanionic ligand) with a high degree of conformational flexibility (Figure 1). In complex **2**, H-bonding directs the polymerization of ferromagnetically coupled tetranuclear cobalt(II) units with an incomplete double cubane core to 1D-single chains. In complex **4**, H-bonding directs the formation of a polynuclear complex in which tetranuclear mixed-valence (Co_2_^II^Co_2_^III^) units are interconnected by a network of H-bonding along the *bc*-axis to produce a beautiful 2D-sheet structure.

**Scheme 1:**
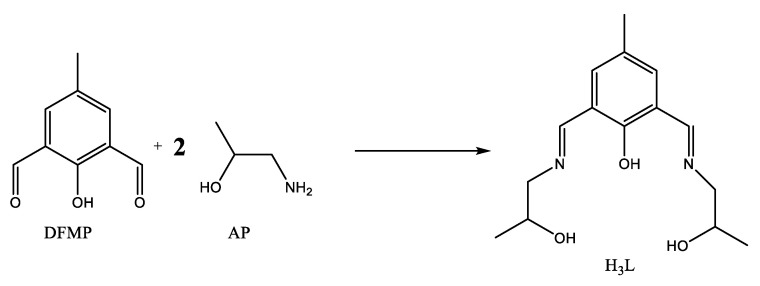
Reactions between DFMP and AP in the presence of Co(II) salts form dinuclear Co(III) complex [Co_2_^III^(H_2_L^−1^)_2_(AP^−1^)(N_3_)](ClO_4_)_2_ (**1**), tetranuclear Co(II) complex [Co_4_(H_2_L^−1^)_2_(µ_3_-1,1,1-N_3_)_2_(µ-1,1-N_3_)_2_Cl_2_(CH_3_OH)_2_]·4CH_3_OH (**2**), and tetranuclear mixed-valence complex [Co_2_^II^Co_2_^III^(HL^−2^)_2_(µ-CH_3_CO_2_)_2_(µ_3-_OH)_2_](NO_3_)_4_·2CH_3_CH_2_OH (**3**) of a double Schiff base ligand H_3_L.

We have carried out a series of reactions between DFMP and 1-amino-2-propanol (AP)/tri(hydroxymethyl)aminomethane (THMAM) in the presence of cobalt(II) salts, CoX_2_ (X = CH_3_CO_2_^−^, NO_3_^−^, Cl, ClO_4_^−^)/NaN_3_/TEA under varied reaction conditions to investigate the effects of various anions (Cl^−^, NO_3_^−^, CH_3_CO_2_^−^, ClO_4_^−^) on the formation of metal clusters with different nuclearities and also on the degree of deprotonation and coordination abilities of the Schiff base ligands (Scheme 1). Earlier [23,52,53,54,55,56,69], we have seen that the nature of the anions, nature of the metal ions, and the reaction conditions (presence/absence of TEA) have remarkable effects on the formation of the ligand, self-assembly of dinuclear, tetranuclear, pentanuclear, hexanuclear, heptanuclear, or decanuclear coordination compounds, in which H-bondings direct the formation of 1D-single chains, 2D-sheets, and 3D-network structures.

**Scheme 2:**
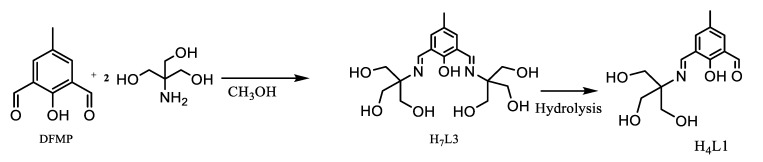
Reactions between DFMP and THMAM in the presence of Co(NO_3_)_2_∙6H_2_O/TEA produces a mixed-valence tetranuclear complex [Co_2_^II^Co_2_^III^(HL1^−2^)_2_(THMAM^−^)_2_][NO_3_]_4_ (**4**) of H_4_L1.

In complex **4**, DFMP and tris(hydroxymethyl)aminomethane (THMAM) were reacted in the presence of Co(NO_3_)_2_·6H_2_O and Gd(CH_3_CO_2_)_3_∙×H_2_O in a 1:2:2.5:2 mole ratio with an intention to get a dinuclear or tetranuclear Co(II)/Gd(III) mixed metal complex of double Schiff base ligand H_7_L3 but instead we got a mixed-valence tetranuclear complex [Co_2_^II^Co_2_^III^(H_2_L1^−2^)_2_(THMAM^−1^)_2_](NO_3_)_4_ (**4**) of a single Schiff base ligand H_4_L1 (Scheme 2) formed from 1 + 1 condensation of DFMP and THMAM. The formation of a single Schiff base ligand (H_4_L1) present in **4** contrary to a double Schiff base ligand (H_7_L3) formed in a pentanuclear copper(II) cluster {[Cu_2_(H_5_L^2^^−^)(μ-N_3_)]_2_[Cu(N_3_)_4_]·2CH_3_OH}_n_, (to the best of our knowledge, the only complex reported with this ligand) [52,55], is presumably due to either cobalt(II) assisted partial hydrolysis of one of the two arms of an initially formed double Schiff base ligand (H_7_L) as we reported earlier [55] in hexanuclear nickel(II) complexes or due to the preferential stereochemical requirements of mixed-valence tetranuclear [Co_2_^II^Co_2_^III^] complex **4** [53,70,71]. Recently, we [53,55,56] and Ray’s group [72] have reported the partial hydrolysis of one of the side arms of H_7_L3/H_5_L2 (Figure 1) or similar double Schiff base ligand (formed by 1 + 2 condensation of DFMP and 2-aminoethanol) in hexanickel (Ni^II^_6_) clusters. Based on present and earlier investigations [52,53,55,56,69] with Schiff base ligands derived from DFMP, we can conclude that the formation of a single Schiff base ligand (H_4_L1) instead of a double Schiff base ligand (H_7_L3) is the result of either metal-catalyzed partial hydrolysis or preferential 1 + 1 condensation in case of hexanuclear Nickel(II) or mixed-valence tetranuclear cobalt complexes due to stereochemical requirements of these metals. 

### 4.2. Description of Structures

#### 4.2.1. [Co_2_^III^(H_2_L^−1^)_2_(AP^−1^)(N_3_)](ClO_4_)_2_ (**1**)

**Figure 2:**
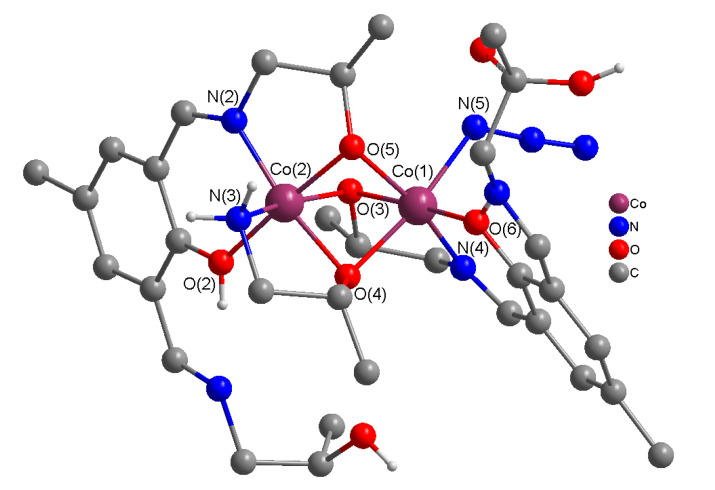
Molecular structure of a dinuclear unit [Co_2_^III^(H_2_L^−1^)_2_(AP^−1^)(N_3_)](ClO_4_)_2_ (**1**) with numbering of atoms in the coordination environment. H atoms are omitted for clarity. Note: In **1**, there is one hydrogen missing because the oxygen atom of the OH group is disordered over two positions. The disordered oxygen atom has been modeled with partial occupancies and is located where the hydrogen atom would be located. We cannot assign a hydrogen atom to that carbon atom due to this issue. Thus, the sum formula is short a hydrogen atom.

Reaction between DFMP and 1-amino-2-propanol (AP) in the presence of Co(ClO_4_)_2_∙6H_2_O/NaN_3_/TEA in methanol (in air) results in the formation of a dinuclear Co(III) complex [Co_2_^III^(H_2_L^−1^)_2_(AP^−1^)(N_3_)](ClO_4_)_2_ (**1**). The cationic core in the structure of **1** is shown in Figure 2. Bond angles and distances relevant to the cobalt(III) coordination core are given in Table S1. Complex **1** crystallizes in triclinic system space group P-1 and is comprised of two Co^3+^ ions, two H_2_L^−1^ ligands, one deprotonated AP^−1^, and one terminal azide ion. H_3_L, potentially a pentadentate trianionic ligand, in **1** behaves as a tridentate (NO_2_) monoanionic (H_2_L^−1^) ligand which is contrary to its behavior as a pentadenate/tetradentate di-anionic ligand in Cu(II) [69], Ni(II) [56], and other cobalt(II) complex (**2**), and mixed-valence Co_2_^II^/Co_2_^III^ complex (**3**). The stereochemistry at Co(1) in an asymmetric dinuclear unit can best be described as a distorted octahedral with phenoxide O, alkoxide O, an azido nitrogen N, and an alkoxide O of AP^−1^ atoms in the equatorial plane and an imine nitrogen of the ligand and an alkoxide O of AP^−1^ in the axial plane. The geometry at Co(2) is defined by phenoxide O, two alkoxide O atoms of the ligands, and an imine N of AP^−1^ atoms in the equatorial plane and an alkoxide O of AP^−1^ and an imine N of the ligand H_2_L^−1^ in the axial plane. The unique feature of this complex is that all distances equatorial and axial are less than 2.00 Å. The other unique feature of this complex is that the metal–metal distance is 2.5430 (11) Å, which is short enough to make Co(III) as seven coordinate, a rare coordination number for cobalt(III). Two Co(III) ions in the dinuclear unit are bridged by three single-atom bridges—two from alkoxide O atoms in the side arms of the ligands (H_2_L^−1^) and one from alkoxide O in AP^−1^, holding two metal centers in close proximity. The sum of the angles in the basal plane of Co(1) and Co(2) are 359.5(2)° and 358.6(2)°, respectively, indicating planar arrangements around these metal centers. The Co-N and Co-O bond distances in the basal plane lie in the ranges 1.848 (6)–1.869 (6) Å and 1.874 (5)–1.943 (5) Å, respectively, which are significantly shorter than the distances in Co(II) complex **2** of this ligand. The Co-N and Co-O bond distances in the axial plane are quite short and lie in the ranges 1.934 (6)–1.939 (7) Å and 1.943 (5)–1.968 (5) Å, respectively. The sum of the angles around the alkoxide bridging O-atoms, O(3), O(4), and O(5) are 312.7 (4)°, 312.8 (4)°, and 310.6 (6)°, respectively, indicating pyramidal distortion at these atoms. 

#### 4.2.2. [Co_4_(H_2_L^−1^)_2_(µ_3_-1,1,1-N_3_)_2_(µ-1,1-N_3_)_2_Cl_2_(CH_3_OH)_2_]·4CH_3_OH (**2**) 

**Figure 3:**
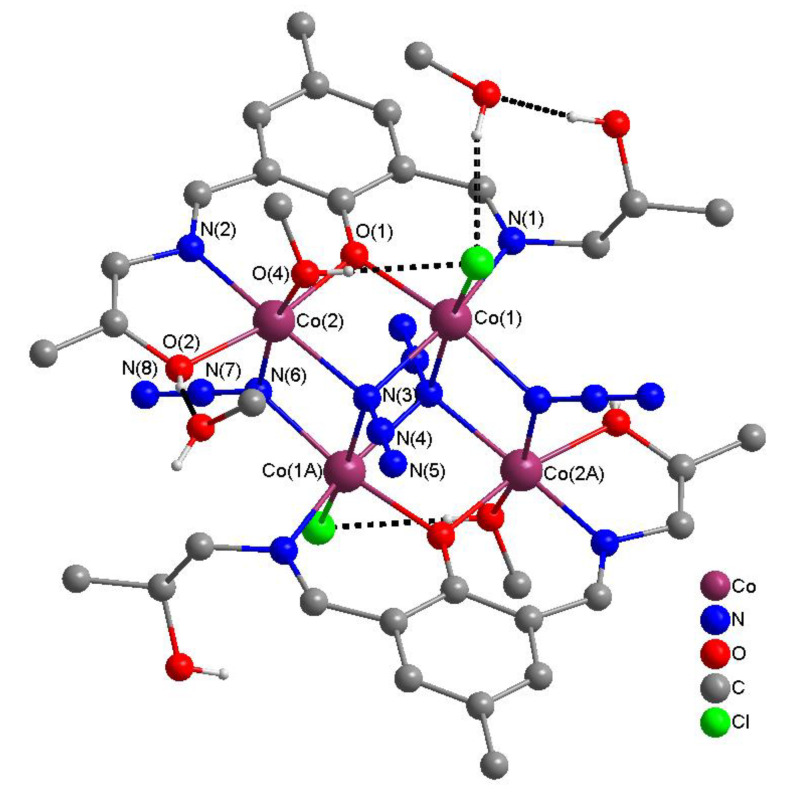
Molecular structure of a neutral centrosymmetric tetranuclear unit [Co_4_(H_2_L^−1^)_2_(µ_3_-1,1,1-N_3_)_2_(µ-1,1-N_3_)_2_Cl_2_(CH_3_OH)_2_]·4CH_3_OH (**2**) with numbering of atoms in the coordination environment. H atoms and solvent molecules are omitted for clarity. Atoms with A in their labels are symmetry generated.

Complex **2** is obtained by reacting DFMP with 1-amino-2-propanol in the presence of CoCl_2_∙6H_2_O/TEA/NaN_3_, crystallizing in the monoclinic system with space group P2_1_/c. Complex **2** has a centrosymmetric tetranuclear Cobalt(II) core [Co_4_(H_2_L^−1^)_2_(µ_3_-1,1,1-N_3_)_2_(µ-1,1-N_3_)_2_Cl_2_(CH_3_OH)_2_]·4CH_3_OH (**2**) in which two imperfect cubanes are fused. The structure of neutral tetranuclear core of **2** is shown in Figure 3 and the relevant bond angles and bond distances for **2** are given in Table S2. In each tetranuclear core, two dinuclear units ([Co_2_(H_2_L^−1^)(µ_3_-1,1,1-N_3_)(µ-1,1-N_3_)Cl(CH_3_OH)]) are interconnected through two triply bridging end-on (EO) μ_3_-N_3_ ions and two doubly bridging EO (μ-N_3_) azide ions. Each dinuclear unit involves one monoanionic tetradenate double Schiff base ligand (H_2_L^−1^), one methanol molecule, one chloride ion, one EO μ-N_3_ ions which provides interdimer bridge, and one EO μ_3_-N_3_ ion which provides intradimer and interdimer bridges. We believe the self-assembly of tetranuclear complex **2** is the result of stereochemical requirements of the cobalt centers. 

In **2**, H_3_L behaves as a tetradentate (N_2_O_2_) monoanionic (H_2_L^1−^) ligand, and one hydroxy group in one side arm of the double Schiff base ligand (H_3_L) remains protonated and uncoordinated. The intradimer and interdimer Co-Co distances of 3.1598 (2) Å and 3.2927 (3) Å, respectively, are much longer compared to **1** (2.543 (11) Å). This is consistent with Co(III) ions in **1** and Co(II) ions in **2**. In each dinuclear unit, two Cobalt(II) ions are bridged by phenoxide oxygen (O(1)) and an azido nitrogen (N(3)) for effective spin-exchange interactions. The sum of the bond angles at μ_-_phenoxide oxygen (O(1)), azide nitrogens [μ-N_3_ (N(6)) and μ_3_-N_3_ (N(3)] are 357.11 (13)°, 357.31 (15)°, and 290.8 (9)°, respectively, and are indicative of slight distortion from planarity at O(1) and N(6) and cubic distortion at N(3) atoms. These bridges can provide effective superexchange interactions between the Co(II) ions in the tetranuclear core. All azides are almost linear (N–N–N = 178.5 (3)–178.6 (3)°). The intradimer bridge angles at the phenoxide oxygen (O(1)) and azide nitrogen (N(3)) are 100.36(8)° and 93.85(9)°, respectively, and provide pathways for antiferromagnetic and ferromagnetic interactions, respectively. The interdimer bridge angles at N(6) (μ-N_3_) and N(3) (μ_3_-N_3_) are 103.36 (9)° and 97.29 (8)–99.68 (9)°, respectively, are indicative of ferromagnetic spin exchange interactions between cobalt centers (vide supra). The stereochemistry at each Cobalt(II) ion is distorted octahedral. The Co-O (2.0468 (19)–2.1410 (19) Å), Co-N (H_2_L^−1^) (2.016 (2)–2.082 (2) Å, Co-N (N_3_^−^) (2.079 (2)–2.248 (2) Å, and Co-Cl (2.4751980 Å) distances are normal and fall in the range reported for octahedral Nickel(II) [56] and Cobalt(II) complexes with this and similar Schiff base ligands. 

**Figure 4:**
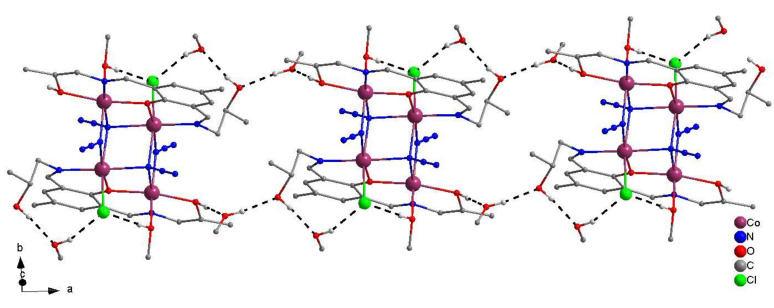
Perspective view of a H-bonding-directed 1D-single chains structure of **2** along the *a*-axis.

H-bonding, which plays a dominating role in the stabilization of proteins and other biomolecules structures, direct the polymerization of the tetranuclear [Co_4_] units producing 1D-single chains (Figure 4), which are not connected in any way to form 2D-sheets or 3D-structures as observed in mixed-valence [Co_2_^II^Co_2_^III^] tetranuclear complex **4**. There are intramolecular H-bonding interactions between the coordinated chloride ion (Cl(1)) and hydroxy group (O(4)) of the coordinated methanol on one side (3.038 Å) and hydroxy group (O(5)) of the uncoordinated methanol (3.114 Å) on the other side, which is further H-bonded (2.706 Å) to the uncoordinated hydroxy group (O(3)) in the side arm of the ligand (H_2_L^−^). The tetranuclear units are interconnected by remarkably strong symmetrical network of hydrogen bonding interactions (2.608–2.664 Å) between hydroxy group (O(6)) of the uncoordinated methanol molecule to the coordinated protonated hydroxy group (O(2)) in the side arm of the ligand (H_2_L^−^) and uncoordinated protonated hydroxy group (O(3)) in the side arm of H_2_L^−^ in the neighboring molecule producing 1D-single chains (Figure 4).

##### 4.2.3. [Co_2_^II^Co_2_^III^(HL^−2^)_2_(µ-CH_3_CO_2_)_2_(µ_3-_OH)_2_](NO_3_)_2_·2CH_3_CH_2_OH (**3**) 

**Figure 5:**
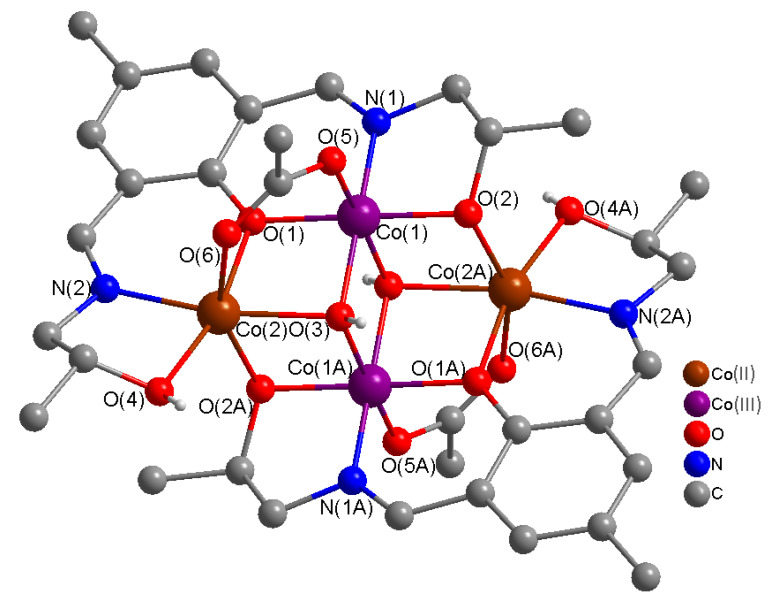
Molecular structure of a centrosymmetric tetranuclear cationic unit [Co_2_^II^Co_2_^III^(HL^−2^)_2_(µ-CH_3_CO_2_)_2_(µ_3-_OH)_2_]^2+^ in (**3**) with numbering of atoms in the coordination environment. H atoms and solvent molecules are omitted for clarity. Atoms with A in their labels are symmetry generated.

The aerobic reaction between DFMP and 1-amino-2-propanol in the presence of Co(NO_3_)_2_·6H_2_O/Gd(CH_3_CO_2_)_3_∙XH_2_O/TEA results in the formation of a mixed-valence tetranuclear complex [Co_2_^II^Co_2_^III^(HL^−2^)_2_(µ-CH_3_CO_2_)_2_(µ_3-_OH)_2_](NO_3_)_2_·2CH_3_CH_2_OH (**3**). It is presumed that the presence of both CH_3_CO_2_^−^ and NO_3_^−^ ions or Gd(III) ion promotes the separation of **3**. In **3**, H_3_L acts as a pentadentate (N_2_O_3_), dianionic ligand (HL^−2^) holding two metal centers in close proximity, whereas in **1** and **2** it behaves as a tridentate monoanionic or tetradentate monoanionic ligand, respectively. A perspective view of **3**, along with relevant atomic labeling, is shown in Figure 5. The selected bond distances and angles relevant to cobalt coordination are given in Table S3. Complex **3** has a cationic centrosymmetric tetranuclear core [Co_2_^II^Co_2_^III^(HL^−2^)_2_(µ-CH_3_CO_2_)_2_(µ_3-_OH)_2_]^+2^ in which two imperfect cubanes are fused and each cobalt (Co^II^/Co^III^) has distorted octahedral stereochemistry. In each dinuclear unit, Co(II) and Co(III) ions are bridged by two single atom bridges (a phenoxide oxygen O(1) (µ-PhO) and a hydroxide oxygen O(3) (µ_3_-OH) and an acetate ions (1,3-CH_3_CO_2_^−^) with a metal–metal separation of 2.9251 (10) Å. Two dinuclear units are interconnected by two triply bridging (µ_3_-OH) hydroxide ions (O(3) and O(3A)) and two alkoxide ions (O(2) and O(2A) in the side arm of the ligand to form mixed-valence tetranuclear cationic core [Co_2_^II^Co_2_^III^(HL^−2^)_2_(µ-CH_3_CO_2_)_2_(µ_3-_OH)_2_]^+2^ with intermetallic separation of 3.0702 (9) Å (Co(1)-Co(2A)).

The coordination environment around Co^III^(1) and Co^II^(2) in the equatorial plane is defined by phenoxy oxygen (O(1)), imino nitrogen (N(1)/N(2)), alkoxide oxygen (O(2)/O(4)), and hydroxide oxygen (O(3)) and in the axial plane by acetate oxygen (O(5)/O(6)) and hydroxide oxygen (O(3A))/alkoxide oxygen (O(2A)), respectively, in a distorted octahedral arrangement. The Co-N and Co-O distances ranging from 1.873 (4) to 2.036 (4) Å and 1.891 (3) to 2.131 (3) Å (Table S3) are very similar to the distances reported for similar mixed-valence cobalt complexes with simiar Schiff base ligands [53,70,71]. The Co-Co distances in the doubly bridged dinuclear and tetranuclear units are in the range 2.9288(16)–3.0702(9) Å which are slightly shorter than the distances reported in similar mixed-valence cobalt complexes [71]. There are two uncoordinated NO_3_^−^ ions per tetranuclear unit in the lattice. In each dinuclear unit, the phenoxide bridge angle (Co(1)-O(1)-Co(2) is 98.35 (15)° and the hydroxide (μ_3_-OH) bridge angle (Co(1)-O(3)-Co(2)) is 94.63(15)° which are significantly smaller than in complex **4**. The bridge angles at the doubly bridging ethanolate oxygen (μ-O) (Co(1)-O(2)-Co(2A)) is 100.86(13) and the triply bridging hydroxide oxygen (μ_3_-O(3)) (Co(1)-O(3)-Co(1A)) and Co(2)-O(3)-Co(1A), which bridge the two dimeric units in the tetranuclear core are 100.12 (16)° and 101.12 (14)°, respectively. The solid angles at the phenoxide (O(1), ethanolate (μ-O(2) and hydroxide oxygen (μ_3_-O(3)) atoms are 359.75 (6)°, 333.66 (6)°, and 295.87 (15)°, respectively, and are indicative of the μ-planar, μ-pyramidal, and μ_3_-cubic arrangements at these bridging centers [70,71].

The oxidation state assignments for the cobalt ions in complex **3** which are color coded (Figure 5) are made using charge considerations, bond valence sums (BVS) calculations, and interatomic distances (Co-N and Co-O). Interatomic distances (Co-O and Co-N) for Co(1) lie in the ranges 1.891–1.927 Å and 1.873 Å, respectively, and those for Co(2) (Co-O and Co-N) lie in the ranges 2.000–2.131 Å and 2.036 Å, respectively. The distances for Co(2) are significantly much longer than Co(1), clearly indicating that Co(1) and its symmetry related Co(1A) are Co^III^ and Co(2) and its symmertry related Co(2A) are Co^II^. The bond valence sums (BVS) calculations using bond length data from the literature [73,74,75,76] were performed on complex **3** to obtain useful and important information about the oxidation state for cobalt ions. BVS values of 3.54 and 2.22 for Co(1) and Co(2), respectively, are typical for Co(III) and Co(II) [73,74,75,76].

#### 4.2.4. [Co_2_^II^Co_2_^III^(H_2_L1^−2^)_2_(THMAM^−1^)_2_](NO_3_)_4_ (**4**)

**Figure 6:**
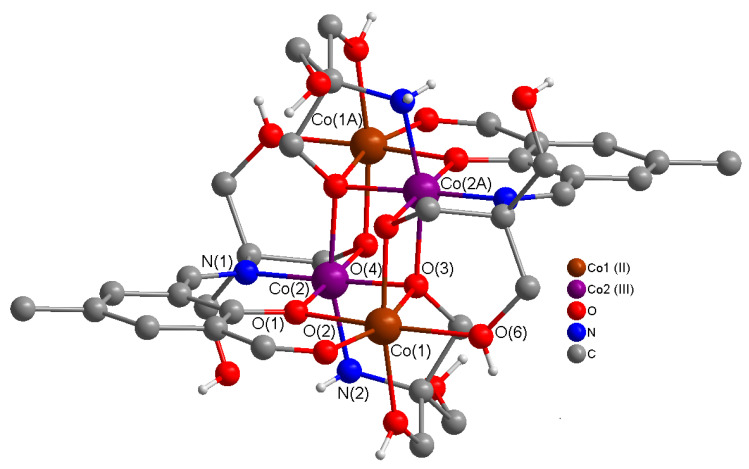
Molecular structure of a cationic centrosymmetric tetranuclear unit [Co_2_^II^Co_2_^III^(H_2_L1^−2^)_2_(THMAM^−1^)_2_]^4+^ (**4**) with numbering of atoms in the coordination environment. H atoms and NO_3_^−^ ions are omitted for clarity. Atoms with A in their labels are symmetry generated.

Aerobic reaction between DFMP and tris(hydroxymethyl)aminomethane (THMAM) in the presence of cobalt(II) nitrate and triethylamine (TEA) result in the formation of a mixed-valence tetranuclear coordination complex, [Co_2_^II^Co_2_^III^(H_2_L1^2^^−^)_2_(THMAM^−^)_2_](NO_3_)_4_ (**4**). The molecular structures of complex **4** consists of dicrete centrosymmetric mixed-valence tetranuclear cationic [Co_2_^II^Co_2_^III^(H_2_L1^2^^−^)_2_(THMAM^2^^−^)_2_]^4+^ unit with two incomplete cubanes fused together (Figure 6). The bond distances and bond angles relevant to the coordination core of cobalt ions are given in Table S4. The basal plane of cobal(1) is defined by a donor set of four oxygen atoms [O(1), O(2), O(3), and O(6A)] from phenoxy oxygen, aldehyde oxygen, and ethanolate oxygen from H_2_L1^2−^, and an alkoxy (μ_3_-O) oxygen atom of THMAM. The axial positons are occupied by ethanolate oxygen (O(4A)) in the side arm of the ligand and an alkoxide oxygen (O(7)) of THMAM in a slightly distorted octahedral arrangement. The basal plane of cobal(2) is defined by a donor set of three oxygen and one nitrogen atoms [NO_3_], from phenoxy oxygen (O(1)), ethanolate oxygen (O(4)), and imino nitrogen (N(1)) from H_2_L1^2-^, and an alkoxy (μ_3_-O(3)) oxygen atom of THMAM. The axial positons are occupied by amino nitrogen (N(2)) and alkoxide oxygen (μ_3_-O(3A)) of THMAM molecules in a slightly distorted octahedral stereochemical arrangement. 

The oxidation state assignments for the cobalt ions are made using charge considerations, BVS calculations, and Co-N and Co-O interatomic distances. Interatomic distances (Co-O) for Co(1) lie in the range 2.014 (3)–2.118 (4) Å and those for Co(2) (Co-N and Co-O) lie in the ranges 1.867 (4)–1.925 (4) Å and 1.877(3)–1.977(3), respectively. The distances for Co(1) are significantly longer than Co(2), clearly indicating that Co(1) and Co(2) have oxidation state of +2 and +3, respectively. Co(1) and its symmetry related Co(1A) and Co(2) and its symmertry related Co(2A) have been assigned Co^II^ and Co^III^, respectively. BVS values of 2.14 and 3.64 for Co(1) and Co(2), respectively, are typical for Co(II) and Co(III) [73,74,75,76].

In **4**, Schiff base ligand (H_4_L1), formed from 1 + 1 condensation of DFMP and THMAM, acts in a pentadenate [NO_4_] dianionic (H_2_L^2−^) capacity binding two metal centers (Co^II^Co^III^), which are linked through triply bridging alkoxy (μ_3_-O(3)) and doubly bridging phenoxy (μ-C_6_H_5_O) oxygen atoms of THMAM and the Schiff base ligand, H_2_L1^2−^, respectively. The two dinuclear units are linked together through two triply bridging alkoxy (μ_3_-O) oxygen atoms of THMAM ligand which act as intra- and inter-dinuclear bridges and two interdinclear, doubly bridging ethanolate (μ-O) oxygen atoms in the side arms of the ligands (H_2_L1^2−^) forming tetranuclear cationic core with non-coordinated NO_3_^−^ ions as counter anions in the crystal lattice which are involved in H-bonding interactions between tetranuclear units forming 2D-sheet structure. 

The Cobalt(II) ions (Co(1) and Co(1A)) have a slightly distorted octahedral [O_6_] arrangement with the Co-O distances ranging from 2.014 (3)–2.118 (4) Å. The Cobalt(III) ions (Co(2) and Co(2A)) in the tetranuclear core exhibit a slightly distorted octahedral [N_2_O_4_] environment with the Co-N distances ranging from 1.867 (4) to 1.925 (4) Å and the Co-O distances ranging from 1.877 (3) to 1.977 (3) Å (Table S4). The Co-N and Co-O distances are very similar to the distances reported for similar mixed-valence cobalt complexes with simiar Schiff base ligands [53,70,71]. The Co-Co distances in the doubly bridged dinuclear and tetranuclear units are in the range 2.9521 (9)–3.055 (1) Å which are slightly shorter than the distances reported in similar mixed-valence cobalt complexes [53]. There are four uncoordinated NO_3_^−^ ions per tetranuclear unit in the lattice. In each dinuclear unit, the phenoxide bridge angle (Co(1)-O(1)-Co(2) is 101.38(13)° and the alkoxide (μ_3_-O(3)) bridge angle (Co(1)-O(3)-Co(2)) is 98.58 (12)°. The bridge angles at the doubly bridging ethanolate oxygen (μ-O(4)) (Co(2)-O(4)-Co(1A)) and the triply bridging ethanoate oxygens (μ_3_-O(3)) (Co(1)-O(3)-Co(2A) and Co(2)-O(3)-Co(2A)) which bridge the two dinuclear units in the tetranuclear core are 101.39 (13)°, 94.96 (12)°, and 98.52(13)°, respectively. The solid angles at the phenoxide (μ-O (1) and ethanolate (μ_3_-O(3), and µ-O(4)) bridges are 358.78 (6)°, 292.06 (6)°, and 326.79 (6)°, respectively, and are indicative of the μ-planar, μ_3_-cubic, and μ-pyramidal arrangements at these bridging centers similar to Co^II^ and mixed-valenec (Co^II^/Co^III^) complexes [53,77,78,79,80,81,82,83,84,85]. 

**Figure 7:**
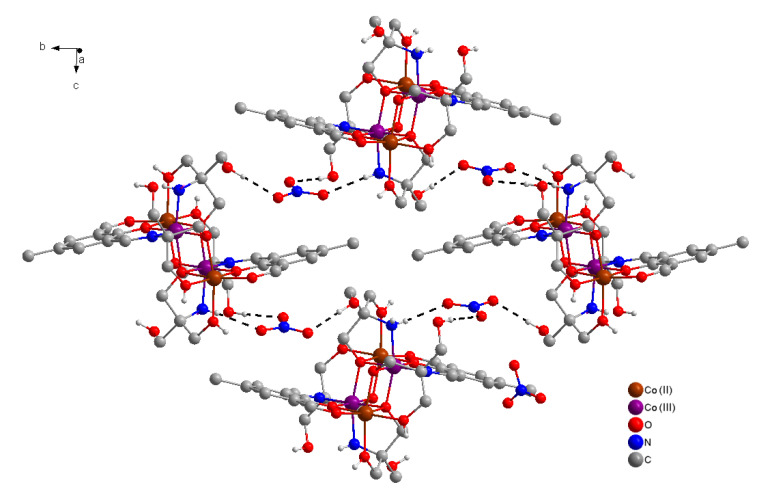
Perspective view of a H-bonding-directed 2D-sheet structure of **4** along the *bc*-axis.

In **4**, a symmetric network of reasonably strong, intermolecular H-bonding interactions (2.765–2.930 Å) between oxygen atoms (O(9), O(10), O(11)) of the uncoordinated NO_3_^−^ ions and the uncoordinated protonated ethanoate oxygen (O(5)) atom in the side arm of the ligand (H_2_L1^2−^) (O(11)–O(5) = 2.805 Å), in one tetranuclear unit and the coordinated amino group (NH_2_) (O(9)–N(2) = 2.930 Å) and the uncoordinated hydroxy (O8)) of THMAM (O(10)–O(8) = 2.765 Å) of the neighboring tetranuclear unit, direct the formation of a beautiful 2D-sheet structure (Figure 7). These H-bonding interactions seem to be responsible for the formation and the stability of complex **4**, as attempts to isolate cobalt complex by reacting Co(CH_3_COOH)_2+_4H_2_O with H4L1 did not succeed.

### 4.3. Magnetic Properties 

**Figure 8:**
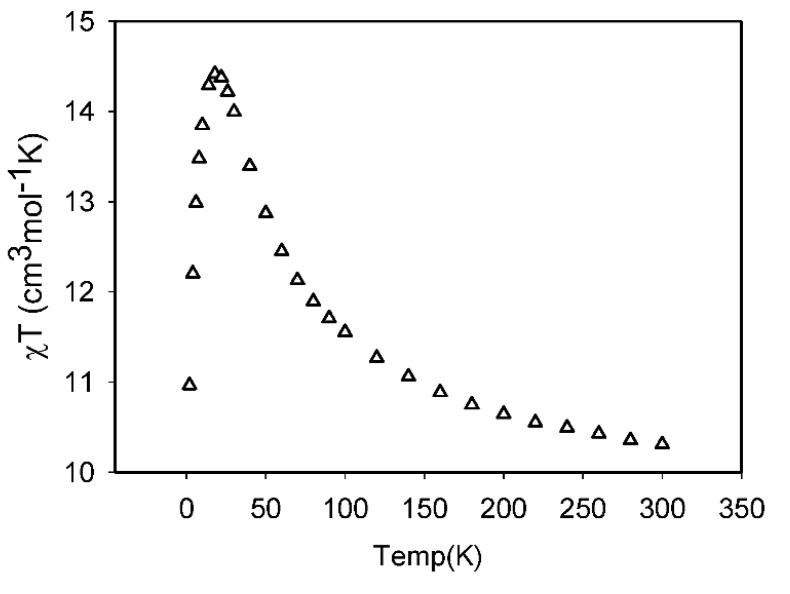
chi.T vs T plot of complex **2**.

The variable temperature magnetic properties of **2** are illustrated in Figure 8 as a plot of chi.T vs T. The characteristic maximum at low temperature suggests intramolecular exchange, dominated by ferromagnetic coupling as reported in ferromagnetically coupled tetranuclear Cobalt(II) complexes involving µ_1,1_-N_3_ and µ-O bridges [86] or pseudo halide bridges [87]. The RT moment per metal is 5.46 muB, which is in the upper range for high spin Co(II). The small rise on lowering temperature is the result of spin orbital coupling effects. While the Co-O-Co angle of 100.4° is consistent with antiferromagnetic exchange in general, the small Co-N-Co angle (93.9°) is in the realm expected for ferromagnetic exchange. In this case, the ferromagnetic component appears to dominate. Fitting of the exchange data to an appropriate Hamiltonian did not give a satisfactory fit, as anticipated for Co(II), due to spin-orbit effects. 

### 4.4. IR and UV–Vis Spectroscopy

In the IR spectra of **1** and **2**, which have terminal azide (N_3_) ions and intra and inter-dimer EO (μ-1,1-N_3_ or μ_3_-1,1,1-N_3_) bridging azide ions, one band is observed at 2227 (1)/2084 cm^−1^, typical for υ_as_ (N_3_). In the IR spectra of **1**–**4**, a band in the region 3445–3333 cm^−1^ and one or two bands in the region 1651–1637 cm^−^^1^ are due to υ(OH) (H_2_O and CH_3_OH) and υ(C=N) of coordinated imine, respectively. In the IR spectra of **1,** three bands in the regions at 3294, 3243, 3142 cm^−1^, and in **4**, one band at 3179 cm^−1^ are due to (υ(NH_2_) of the coordinated amino group of AP^−^^1^and THMAM^−^^1^, respectively. 

In the UV–Vis spectra of **1**–**2** compounds, a strong band at 430–390 nm and a high energy band or shoulder at 320–270 nm are assigned to metal–azide and metal–ligand charge transfer transitions. In the visible spectra of the **1**–**4** complexes, one to three broad bands/shoulders in the region 680–525 nm are due to d–d transitions. 

## 5. Conclusions

In this publication, we have reported one-pot self-assembly and structural characterization of four new Cobalt(III) (**1**), Cobalt(II) (**2**), and mixed-valence (Co^II^/Co^III^) (**3** and **4**) complexes with two Schiff base ligands: H_3_L (double Schiff base ligand) and H_4_L1 (single Schiff base ligand), with a very high degree of conformational flexibility and potential to coordinate in a convergent and a divergent fashions. In complexes **2** and **4**, H-bonding directs the polymerization of neutral tetranuclear Cobalt(II) units [Co_4_(H_2_L^−1^)_2_(µ_3_-1,1,1-N_3_)_2_(µ-1,1-N_3_)_2_Cl_2_(CH_3_OH)_2_]·4CH_3_OH (**2**) and tetranuclear mixed-valence cationic units [Co_2_^II^Co_2_^III^(H_2_L1^−2^)_2_(THMAM^−1^)_2_](NO_3_)_4_ (**4**) to 1D-single chains and 2D-sheet structures, respectively. The uncoordinated/coordinated protonated hydroxy groups in the side arms of the ligands are involved in the intramolecular and intermolecular H-bonding interactions with uncoordinated/coordinated methanol molecules/NO_3_^−^ ions to generate polymeric complexes with 1D-single chains (**2**) and 2D-sheet (**4**) structures. Depending upon the stereochemical requirements of Cobalt(II)/Cobalt(III) centers in dinuclear or tetranuclear units, the azide ions act as intradimer/interdimer EO double (µ-1,1-N_3_) or triple bridges (µ_3_-1,1,1-N_3_), carboxylate as a double bridge, hydroxide ion as a triple bridge (µ_3_-1,1,1-OH). In these complexes, H_3_L acts as a tridentate (NO_2_) monoanionic (**1**), tetradentate (N_2_O_2_) monoanionic (**2**), and pentadentate (N_2_O_3_) dianionic ligand. In complex **4**, H_4_L1 behaves as a pentadentate (NO_4_) dianionic ligand. Further studies to procure coordination clusters of 3d, 4f, and mixed metal (3d–4f) of double Schiff base (H_7_L3) and other similar ligands are in progress.

## Figures and Tables

**Table 1:** Summary of crystallographic data for compounds **1**–**4.**

Compound	1	2	3	4
Empirical formula	C_33_H_50_Cl_2_Co_2_N_8_O_15_	C_36_H_60_Cl_2_Co_4_N_16_O_12_	C_38_ H_60_Co_4_N_6_O_20_	C_34_H_50_Co_4_N_8_O_28_
M	986.56	1215.62	1156.64	1254.54
Crystal System	triclinic	monoclinic	triclinic	monoclinic
Space group	P-1	P2_1_/c	P-1	P2_1_/c
a/Å	12.976(7)	12.3537(13)	9.594(3)	10.9951(12)
b/Å	13.417(7)	12.0935(13)	11.947(4)	18.435(2)
c/Å	13.652(7)	18.306(2)	12.238(4)	14.5466(16)
α/°	77.064(9)	-	94.242(5)	-
β/°	73.785(9)	98.075(2)	110.439(5)	111.377(2)
γ/°	85.693(9)	-	104.116(4)	-
*V/*Å^3^	2224(2)	2707.8(5)	1255.1(7)	2745.7(5)
ρ_calcd_ (g cm^−3^)	1.473	1.491	1.530	1.517
*T/*K	180	105.15	105.15	190
*Z*	2	2	1	2
μ/mm^−1^	0.938	1.370	1.377	1.277
Crystal size (mm)	0.20 × 0.15 × 0.10	0.41 × 0.25 × 0.10	0.2 × 0.2 × 0.1	0.20 × 0.2 × 0.10
Reflections collected:				
Total	17942	21211	9624	21677
Unique	7850	4798	4410	4858
*R_int_*	0.0973	0.0256	0.1010	0.0668
Final *R_1_*, *wR_2_*	0.0806, 0.1931	0.0334, 0.0929	0.0533, 0.1388	0.0539, 0.1262

R_1_ = Σ[│*F_0_*│−│*F_c_*│]/Σ│*F_0_*│, wR_2_ = [Σ[w(│*F_0_*│^2^ − │*F_c_*│^2^)^2^]/Σ[w(│*F_0_*│^2^)^2^]]^1/2^, R = Σ||Fo| − |Fc||/Σ|Fo|, Rw = [Σw (|Fo| − |Fc|)^2^/Σw Fo^2^]^1/2^.

## Supplementary Materials

The following are available online at https://www.mdpi.com/1996-1944/13/23/5425/s1, Table S1–S4: combined Co checkcif report, combined Co structures 2019.
