# Supplementary material for: One-Pot Self-Assembly of Dinuclear, Tetranuclear, and H-Bonding-Directed Polynuclear Cobalt(II), Cobalt(III), and Mixed-Valence Co(II)/Co(III) Complexes of Schiff Base Ligands with Incomplete Double Cubane Core
Source: Materials (Basel). 2020 Nov 28;13(23):5425. doi: 10.3390/ma13235425 (PMC7730246; doi:10.3390/ma13235425)
Supplement: Supplementary file 1 [file materials-13-05425-s001.pdf]

materials

One-Pot Self-Assembly of Dinuclear, Tetranuclear, and H-Bonding-Directed Polynuclear Cobalt(II), Cobalt(III), and Mixed-Valence Co(II)/Co(III) Complexes of Schiff Base Ligands with Incomplete Double Cubane Core

Santokh S. Tandon [1,2,*], Neil Patel [2], Scott D. Bunge [2], Esther C. Wang [2], Rachel Thompson [2] and Laurence K. Thompson [3]

[1] Department of Chemistry and Biochemistry, Kent State University - Salem Campus, Salem, OH 44460, USA
[2] Department of Chemistry and Biochemistry, Kent State University, Kent, OH 44242, USA; neilnpatel89@gmail.com (N.P.); sbunge@kent.edu (S.D.B.); ewang2@kent.edu (E.C.W.); rthoma17@kent.edu (R.T.)
[3] Department of Chemistry, Memorial University, St. John's, NL A1B 3X7, Canada; laurie.thomp@gmail.com
* Correspondence: standon@kent.edu

Table S1. Bond lengths [Å] and angles [°] for **1**.

Bond Lengths	[Å]	Bond Angles			[°]
		N4	Co1	O3	88.5(2)
		N4	Co1	O6	95.5(3)
		O3	Co1	O5	80.7(2)
		O6	Co1	O5	94.8(2)
		N4	Co1	O5	167.5(2)
		N5	Co1	O4	171.3(3)
		O6	Co1	O3	173.6(2)
Co(1)-N(4)	1.848(6)				
Co(1)-N(5)	1.939(7)	N2	Co2	N3	93.3(3)
Co(1)-O(3)	1.897(5)	N2	Co2	O3	95.5(2)
Co(1)-O(4)	1.968(5)	O4	Co2	N3	89.0(2)
Co(1)-O(5)	1.943(5)	O4	Co2	O3	80.8(2)
Co(1)-O(6)	1.874(5)	N2	Co2	O4	170.8(3)
Co(2)-N(2)	1.869(6)	N3	Co2	O3	167.0(2)
Co(2)-N(3)	1.934(6)	O5	Co2	O2	176.3(2)
Co(2)-O(2)	1.907(5)				
Co(2)-O(3)	1.943(5)	Co1	O3	Co2	82.93(18)
Co(2)-O(4)	1.890(5)	C20	O3	Co1	108.9(4)
Co(2)-O(5)	1.881(5)	C20	O3	Co2	120.9(4)
Co(1)-Co(2)	2.543	Co2	O4	Co1	82.45(19)
		C17	O4	Co1	120.6(4)
		C17	O4	Co2	109.8(4)
		Co2	O5	Co1	83.36(19)
		C14	O5	Co1	118.7(4)
		C14	O5	Co2	108.6(4)

Table S2. Bond lengths [Å] and angles [°] for **2.**

Bond Lengths	[Å]	Bond Angles	[°]
		N(6A)-Co(1)-N(1)	96.98(9)
		N(6A)- Co(1)-N(3)	91.96(9)
		O(1)-Co(1)-N(1)	87.91(8)
		O(1)-Co(1)-N(3)	81.82(8)
		O(1)-Co(1)-N(6A)	169.59(8)
		N(1)-Co(1)-N(3)	167.06(8)
Co(1)-N(1)	2.082(2)	N(3A)-Co(1)-Cl(1)	168.56(6)
Co(1)-N(3)	2.188(2)		
Co(1)-N(3A)	2.248(2)	O(2)-Co(2)-N(3)	106.43(8)
Co(1)-N(6A)	2.079(2)	N(2)-Co(2)-O(1)	91.39(9)
Co(1)-O(1)	2.0674(17)	N(2)-Co(2)-O(2)	79.17(9)
Co(1)-Cl(1)	2.4751(8)	O(1)-Co(2)-N(3)	83.55(8)
Co(2)-N(3)	2.137(2)	O(1)-Co(2)-O(2)	168.99(8)
Co(2)-N(6)	2.118(2)	N(6)-Co(2)-O(3)	165.63(8)
Co(2)-N(2)	2.016(2)	N(2)-Co(2)-N(3)	172.05(9)
Co(2)-O(1)	2.0468(19)		
Co(2)-O(3)	2.1410(19)	Co(2)-N(3)-Co(1)	93.85(9)
Co(2)-O(2)	2.131(2)	Co(1)-N(3)-Co(1A)	99.66(9)
Co(1)-Co(2)	3.160	Co(2)-N(3)-Co(1A)	97.29(8)
Co(1)-Co(2A)	3.293		
		Co(1A)-N(6)-Co(2)	103.36(9)
		N(7)-N(6)-Co(1A)	130.40(19)
		N(7)-N(6)-Co(2)	123.55(18)
		Co(2)-O(1)-Co(1)	100.36(8)
		C(3)-O(1)-Co(1)	130.73(17)
		C(3)-O(1)-Co)(2)	126.02(16)

Table S3. Bond lengths [Å] and angles [°] for **3.**

Bond Lengths	[Å]	Bond Angles	[°]
		O(2)-Co(1)-O(3)	95.97(14)
Co(1)-Co(1A)	2.9288(16)	O(1)-Co(1)-O(3)	85.98(14)
Co(1)-Co(2)	2.948	N(1)-Co(1)-O(2)	84.84(15)
Co(2)-Co(1A)	3.071	N(1)-Co(1)-O(1)	92.96(16)
Co(1)-O(2)	1.891(3)	N(1)-Co(1)-O(3)	177.08(16)
Co(1)-O(1)	1.895(3)	O(3A)-Co(1)-O(5)	170.67(15)
Co(1)-O(5)	1.908(3)	O(2)-Co(1)-O(1)	174.43(14)
Co(1)-O(3A)	1.892(3)	O(1)-Co(2)-O(3)	79.37(13)
Co(1)-O(3)	1.928(3)	O(1)-Co(2)-N(2)	88.39(15)
Co(1)-N(1)	1.873(4)	O(3)-Co(2)-O(4)	112.36(14)
Co(2)-O(2A)	2.089(3)	N(2)-Co92)-O(4)	79.75(16)
Co(2)-O(1)	2.000(3)	N(2)-Co(2)-O(3)	167.75(16)
Co(2)-O(4)	2.099(4)	O(2A)- Co(2)-O(6)	156.22(11)
Co(2)-O(6)	2.131(3)	O(1)-Co(2)-O(4)	165.74(13)
Co(2)-O(3)	2.081(3)	Co(1)-O(2)-Co(2A)	100.86(13)
Co(2)-N(2)	2.036(4)	C(10)-O(2)-Co(1)	106.7(3)
		C(10)-O(2)-Co(2A)	126.1(3)
		Co(1)-O(1)-Co(2)	98.35(15)
		C(5)-O(1)-Co(1)	129.4(3)

		C(5)-O(1)-Co(2)	132.0(3)
		Co(1A)-O(3)-Co(1)	100.12(16)
		Co(1A)-O(3)-Co(2)	101.12(14)
		Co(1)-O(3)-Co(2)	94.63(15)

Table S4. Bond lengths [Å] and angles [°] for **3**.

**Bond Lengths**	**[Å]**	**Bond Angles**	**[°]**
		O(1)-Co(1)-O(3)	75.84(11)
		O(2)-Co(1)-O(1)	88.29(12)
		O(2)-Co(1)-O(6A)	100.20(14)
		O(3)-Co(1)-O(6A)	95.70(14)
		O(4A)-Co(1)-O(7)	168.41(15)
		O(1)-Co(1)-O(6A)	171.51(14)
		O(2)-Co(1)-O(3)	162.99(12)
Co(1)-O(1)	2.049(3)		
Co(1)-O(2)	2.036(3)	O(1)-Co(2)-O(3)	84.07(12)
Co(1)-O(3)	2.108(3)	O(4)-Co(2)-O(3)	94.78(12)
Co(1)-O(4A)	2.014(3)	N(1)-Co(2)-O(1)	95.49(14)
Co(1)-O(6A)	2.118(4)	N(1)-Co(2)-O(4)	85.59(14)
Co(1)-O(7)	2.037(4)	N(2)-Co(2)-O(3A)	165.50(16)
Co(2)-Co(2A)	2.9522(11)	O(4)-Co(2)-O(1)	177.50(13)
Co(2)-O(1)	1.897(3)	N(1)-Co(2)-O(3)	178.16(14)
Co(2)-O(3A)	1.977(3)		
Co(2)-O(3)	1.919(3)	Co(2)-O(1)-Co(1)	101.38(13)
Co(2)-O(4)	1.877(3)	C(5)-O(1)-Co(1)	130.7(3)
Co(2)-N(1)	1.867(4)	C(5)-O(1)-Co(2)	126.7(3)
Co(2)-N(2)	1.925(4)		
		Co(2A)-O(3)-Co(1)	94.96(12)
		Co(2)-O(3)-Co(1)	98.58(12)
		Co(2)-O(3)-Co(2A)	98.52(13)
		C(15)-O(3)-Co(1)	117.2(3)
		C(15)-O(3)-Co(2)	108.3(3)
		C(15)-O(3)-Co(2A)	133.2(3)
		Co(2)-O(4)-Co(1A)	101.39(13)
		C(11)-O(4)-Co(1A)	114.6(3)
		C(11)-O(4)-Co(2)	110.8(3)

Publisher's Note: MDPI stays neutral with regard to jurisdictional claims in published maps and institutional affiliations.

© 2020 by the authors. Submitted for possible open access publication under the terms and conditions of the Creative Commons Attribution (CC BY) license (http://creativecommons.org/licenses/by/4.0/).
